# Effects of safe handling education on cognition, compliance and stress handling of antineoplastic drugs in clinical nurses

**DOI:** 10.1002/nop2.1626

**Published:** 2023-02-09

**Authors:** Eun‐Mi Jun, Se‐Won Kang

**Affiliations:** ^1^ Department of Nursing Pai Chai University Daejeon South Korea; ^2^ Department of Nursing Dongseo University Busan South Korea

**Keywords:** antineoplastic drug, cognition, compliance, education, stress

## Abstract

To evaluate the effects of safe‐handling education on the cognition, practice and stress handling of antineoplastic drugs in clinical nurses. This study uses a quasi‐experimental, non‐equivalent control group pre‐test and post‐test design. The experimental and control groups had 30 nurses each, who handled antineoplastic drugs from three institutions. This study examines the safe handling of antineoplastic drugs six times, for two hours each over two weeks. To verify the homogeneity of the experimental and control groups and the effectiveness of safe‐handling education about antineoplastic drugs, a chi‐square test and independent samples t‐test were performed. The results were statistically significant in both groups (cognition [*t* = 6.84, *p* < 0.001], practice [*t* = 5.86, *p* < 0.001], and the stress of handling antineoplastic drugs [*t* = 5.15, *p* < 0.001]). Education on ways to safely handle antineoplastic drugs improves cognition, practice and stress handling of these drugs; moreover, proper education minimizes exposure.

## 1 INTRODUCTION

As the use of anticancer or antineoplastic drugs has increased owing to the recent increase in cancer incidences, the literature's interest in antineoplastic drugs' toxicity has expanded. Now, studies examine not only patients' but also medical personnel's exposure. As antineoplastic drugs inhibit tumour growth by disrupting cell division and killing fast‐growing cells, they may also be carcinogenic, mutagenic and teratogenic. This can cause subsequent cancer in patients receiving antineoplastic drugs and ongoing side effects, such as immunosuppression, impaired reproductive function and birth defects in pregnant women (Crickman, 2017; Menonna‐Quinn et al., 2019; Park et al., 2019).

The side effects of antineoplastic drugs affect not only patients receiving the drugs but also hospital workers such as nurses and pharmacists who prepare or administer them. Healthcare workers who have treated patients with anticancer drugs for extended period of time have reported experiencing dermatitis and liver damage as side effects of antineoplastic drugs (Shu et al., 2020). Nurses treated with antineoplastic drugs experience birth defects and foetal death during early pregnancy (Challinor et al., 2020; DeJoy et al., 2017). In addition to direct contact with anticancer drugs, indirect exposure through the inhalation of and contact with bodily fluids or excreta of patients taking anticancer drugs can also occur (Coyne et al., 2019).

Nurses are mainly exposed to anticancer drugs through their eyes, digestive tract, respiratory mucosa, and skin during drug preparation. Examples of potential moments of exposure include handling bottles of, diluting, or filling a syringe with medication (Friese et al., 2020; Yun & Park, 2016). They may be exposed when handling urine, faeces, sweat, vomit and blood discharged from patients receiving anticancer drugs. Additionally, they can be exposed through the air, as small particles that are contaminated with anticancer drugs enter a space (Friese et al., 2015; Graeve et al., 2017). Pirot et al. (2022) report that blood contamination occurs for both caregivers and non‐caregivers, and that protective precautions should be taken when handling anticancer drugs. Additionally, according to the survey results of Boiano et al. (2014), a lack of protection and inadequate decontamination procedures cause surface infection, conveying the necessity of following safe handling guidelines.

## 2 BACKGROUND

The Korean Society for Oncology Nursing enacted safety management guidelines for anticancer drugs in 2008, stipulating specific safety practices for minimizing exposure to anticancer drugs and emphasizing education for its implementation (Korean Oncology Nursing Society, 2008). In 2017, safety practice guidelines for oral anticancer drugs were established and distributed among all members of the Society (Park et al., 2017). The Oncology Nursing Society and American Society of Clinical Oncology established anticancer drug practice guidelines, suggesting specific safety management guidelines. They further established a precedence for periodic revisions (Blecher et al., 2003; Polovich, 2011).

Korea's hospital certification evaluation has been in place since 2010. In it, standards for the management of harmful environments and substances, preparation of anticancer drugs and administration and disposal of anticancer drugs are investigated to determine whether the safe management of anticancer drugs has been implemented (Chang et al., 2016). However, confusion over certain cases remains because specific performance standards have not yet been established. Therefore, more detailed guidelines are required.

Survey studies on the knowledge and practice of safe management of anticancer drugs and exposure studies have been conducted (Kim et al., 2016; Park & Kim, 2018; Yun & Park, 2016). An experimental study related to knowledge and performance was conducted for intervention studies related to anticancer safety management education (Chang et al., 2016). As the number of educational intervention studies is minimal and the evaluation of effectiveness has mainly focused on knowledge and performance, very few studies on other variables have been carried out. To date, there has been no verification of the effects on the variables related to the stress of anticancer drug treatment.

Therefore, in this study we analysed the effects of education on the safe handling of antineoplastic drugs, based on guidelines developed by the Occupational Safety and Health Administration (OSHA) (United States Department of Labor, 2012) on cognition, compliance and the stress of handling anticancer drug.

### 2.1 Research question

This study aimed to evaluate the effects of safe handling education on the cognition, compliance and stress handling of antineoplastic drugs in clinical nurses. The following hypothesis was proposed:

Hypothesis 1 *The experimental group with the safe handling education of antineoplastic drugs will have a higher score on cognition than the control group*.

Hypothesis 2 *The experimental group with the safe handling education of antineoplastic drugs will have a higher score on compliance than the control group*.

Hypothesis 3 *The experimental group with the safe handling education of antineoplastic drugs will have a lower score on stress handling of antineoplastic drugs than the control group*.

## 3 METHOD

### 3.1 Design

This study used a quasi‐experimental non‐equivalent control group pre‐test and post‐test design to verify the effects that safe handling education of antineoplastic drugs has on cognition, compliance and stress handling (Table 1).

**TABLE 1:** Experimental design

Group	Pre‐test	Experimental treatment	Post‐test (after six weeks)
Experimental group (EG)	EG1	X	EG2
Control group (CG)	CG1		CG2

*Note*: Experimental treatment: safe handling education, total 12 h—six times for two weeks.

### 3.2 Participants

The study participants were ward nurses dealing with anticancer drugs at general hospitals with more than 300 beds. The inclusion criterion was that the nurses must have been working with anticancer drugs for the past month.

In the experimental group, 30 nurses from Hospital A in city D were conveniently sampled. The control group was randomly assigned to two hospitals (hospitals X and Y) to prevent transmission of experimental effects between participants during the study process. Thirty nurses from Hospital X and Y participated in the study as a control group.

The subjects utilized the G‐power 3.1 program. For the mean difference test of the two groups, the significance level was set at 0.05, the power of a test (1‐β) was 0.80, and the result was calculated as an effect size of 0.80. A 52‐person minimum was required, with 26 people in each group. Considering the dropout rate of 10%, 30 people were assigned to the experimental group and the control group each. There were no dropouts in the final data; therefore, a total of 60 people were analysed.

### 3.3 Study progress

#### 3.3.1 Experimental treatment

At Hospital A, the participants first responded to three kinds of questionnaires on cognition, compliance and stress of treatment with anticancer drugs. Next, they received safe handling education for two weeks from 16 May to 30 May, 2019.

The education process took 12 h and happened six times over two weeks. The lectures and practices were combined. Lectures were conducted with the whole group present, and the practice was conducted in groups of four to five people at a time. The post‐test was conducted six weeks after the education sessions.

#### 3.3.2 Composition and application of education content

The contents of the safe handling education this study used were based on the Anticancer Safety Management Principles of OSHA (Yodaiken & Bennett, 1986). and the safety management guidelines for anticancer drugs published by the Korean Society for Oncology Nursing (Korean Oncology Nursing Society, 2008; Park et al., 2017).

The educational program of this study includes the following: importance of anticancer drug safety management, major exposure routes for administrators of anticancer drugs; potential health problems due to exposure; safety management tips when exposed to anticancer drugs and safety management during anticancer drug preparation; drug administration and disposal; health evaluation; and supervision. The lectures and practice took place for one hour each. This approach was finalized after consultation with an oncology doctor, head nurse, staff nurse, and an advanced nurse practitioner in the anticancer ward.

The lectures were conducted by two researchers, using PowerPoints, and handouts were distributed to the participants. The practice was conducted through demonstrations and exercises, and were led the by two researchers and two advanced oncology nurse practitioners (Table 2).

**TABLE 2:** Contents of safe handling education

Class	Lesson contents		Learning methods
1st	Lecture	The importance of antineoplastic safety management, major road to application	Lecture PPT, Discussion
	Practice	Biological Safety Cabinet: BSC Private Protective Equipment: PPE	Demonstration Practice
2nd	Lecture	Potential health problems due to exposure to antineoplastic drugs, anticancer safety management tips	Lecture PPT, Discussion
	Practice	Preparation of light‐ready antineoplastic drugs, injectable antineoplastic drugs	Demonstration Practice
3rd	Lecture	Safety management when preparing antineoplastic drugs	Lecture PPT, Discussion
	Practice	Oral administration, intravenous administration of antineoplastic drugs	Demonstration Practice
4th	Lecture	Safety management when administering antineoplastic drugs	Lecture PPT, Discussion
	Practice	Oral administration, intravenous administration of antineoplastic drugs	Demonstration Practice
5th	Lecture	Safety management when disposing of antineoplastic drugs	Lecture PPT, Discussion
	Practice	Muscle, underlying injections of antineoplastic drugs	Demonstration Practice
6th	Lecture	Health Assessment and Supervision	Lecture PPT, Discussion
	Practice	Treatment of excrement, secretions and linen Disposal and breakage of antineoplastic drugs	Demonstration Practice

### 3.4 Measurement

#### 3.4.1 Cognition of safe handling antineoplastic drug

Choi et al. (2004) developed a measurement of the cognition of the safe handling of antineoplastic drugs in response to OSHA's recommendations, and that is the measurement utilized in this study. It consists of 16 items about preparation, 14 items on administration and 5 items on disposing of the anticancer agent. Each question was rated on a 4‐point Likert scale; thus, the higher the score, the higher the cognitive degree. Permission was obtained from the developer to use this tool.

Using Cronbach's ⍺ value, the reliability of this tool was 0.92 for preparation, 0.87 for administration and 0.77 for disposal of the anticancer agent (Choi et al., 2004). In this study, it was 0.79 for preparing, 0.90 for administration and 0.79 for disposal of the anticancer agent.

#### 3.4.2 Compliance of safe handling antineoplastic drug

The same tool used in the measurement of the cognition of safe handling antineoplastic drugs developed by Choi et al. (2004) was used to measure compliance of safe handling of antineoplastic drugs. Permission was obtained from the developer to use this tool.

In the study by Choi et al. (2004), the reliability of the tool via the Cronbach's ⍺ value was 0.89 for preparation, 0.84 for administration and 0.60 for disposal of the anticancer agent. In this study, it was 0.79 for preparation, 0.80 for administration and 0.71 for disposal of the anticancer agent.

#### 3.4.3 Stress handling antineoplastic drugs

A tool for measuring the stress of handling of antineoplastic drugs was used by Kim (1996). For this study, 15 items were rated on a 4‐point Likert scale, indicating that the higher the score, the higher the degree of stress handling of antineoplastic drugs. Permission was obtained from the developer to use this tool. The reliability of the tool is 0.90 (Kim, 1996), in this study, it was 0.91.

### 3.5 Data analysis

The collected data were analysed using PASW Statistics 18.0, according to the following statistical methods:

- Homogeneity of cognition, compliance and the stress handling of antineoplastic drugs were analysed using chi‐squares and independent samples *t*‐tests.
- The difference in the safe handling education of antineoplastic drugs between the experimental and control groups was verified by using the independent samples *t*‐test and analysis of covariance (ANCOVA).

### 3.6 Ethical considerations

This study was conducted under the guidance of the university's ethics committee. The researchers visited selected hospitals in person, explained their research purposes and methods to the nursing department and director, and obtained consent for the study process. Furthermore, they explained the study's purpose and methods to the participants and obtained their written consent. The researchers informed participants of their ability to participate and conveyed that the latter would remain anonymous. After the post‐test, the control group was provided with safe handling education of antineoplastic drugs through group education.

## 4 RESULT

### 4.1 Homogeneity of participants' characteristics

The demographics of the experimental and control groups were compared and the variables were: age, sex, marital status, position, workplace, clinical experience, safety education experience of antineoplastic drugs, inhaled exposure to antineoplastic drugs, and skin contact exposure to antineoplastic drugs. No significant differences were observed between the two groups about these variables (Table 3).

**TABLE 3:** Homogeneity test of characteristics between the experimental and control groups (*n* = 60)

Variables		Total	Experimental group (*n* = 30)	Control group (*n* = 30)	χ^2^/t	*p*
		*n* (%)	*n* (%)	*n* (%)		
Age(year)	(mean ± SD)		31.0 ± 7.36	30.52 ± 7.00	0.21	0.840
Gender	Female	60 (100.0)	30 (100.0)	30 (100.0)		
Marital status	Unmarried Married	45 (75.0) 15 (25.0)	22 (36.7) 8 (13.8)	23 (38.3) 7 (11.7)	0.72	0.784
Position	Nurse Staff nurse	53 (88.3) 7 (11.7)	26 (43.3) 4 (6.7)	27 (45.0) 3 (5.0)	1.67	0.431
Working place	Surgical ward Medical ward	21 (35.0) 39 (65.0)	15 (25.0) 15 (25.0)	6 (10.0) 24 (50.0)	5.93	0.291
Clinical experience	<1 1–3 >4	11 (18.3) 27 (45.0) 22 (36.6)	6 (10.0) 13 (21.7) 11 (18.3)	5 (8.3) 14 (23.3) 11 (18.3)	1.02	0.411
Safety Education experience	Yes No	31 (51.7) 29 (48.3)	14 (23.3) 16 (26.7)	17 (28.3) 13 (21.7)	0.60	0.613
Inhaled exposure	Yes No	27 (45.0) 33 (55.0)	10 (16.7) 20 (33.3)	17 (28.3) 13 (21.7)	3.30	0.122
Skin contacted exposure	Yes No	42 (70.0) 18 (30.0)	19 (31.7) 11 (18.3)	23 (38.3) 7 (11.7)	1.27	0.401

### 4.2 Homogeneity between two groups of dependent variables

Before the education sessions, an analysis of the homogeneity of cognition, compliance and stress handling of antineoplastic drugs between the experimental and control groups (Table 4) took place. There were no statistically significant differences between the two groups. However, in the sub‐area of cognition about safe handling education, there was a difference in the cognition scores for the disposal of anticancer agents (*t* = −2.04, *p* = 0.046).

**TABLE 4:** Homogeneity test of outcome variables between the experimental and control groups (*n* = 60)

Outcome Variables	Experimental group (*n* = 30)	Control group (*n* = 30)	*t*	*p*
	Mean ± SD	Mean ± SD		
Cognition of safe handling of antineoplastic drugs				
Total score	102.77 ± 17.23	110.55 ± 16.68	−1.76	0.083
Preparing antineoplastic drugs	46.50 ± 9.09	50.72 ± 9.10	−1.78	0.080
Administering antineoplastic drugs	42.60 ± 7.56	44.10 ± 7.27	−0.78	0.437
Disposing of antineoplastic drugs	13.67 ± 3.64	15.47 ± 3.17	−2.04	0.046
Compliance of safe handling of antineoplastic drug				
Total score	86.75 ± 9.50	81.93 ± 9.47	1.39	0.176
Preparing antineoplastic drugs	37.16 ± 4.91	34.80 ± 7.00	1.15	0.257
Administering antineoplastic drugs	38.62 ± 5.20	37.33 ± 3.68	0.81	0.418
Disposing of antineoplastic drugs	12.00 ± 1.92	11.17 ± 1.76	1.51	0.137
The stress handling of antineoplastic drugs				
Total score	46.57 ± 8.65	47.47 ± 11.97	−0.43	0.672

### 4.3 Hypothesis test

#### 4.3.1 Hypothesis 1

Hypothesis 1 states that “the experimental group with safe handling education of antineoplastic drugs would have a higher score of cognition than the control group.” The results were statistically significant (*t* = 6.84, *p* < 0.001) (Table 5), and Hypothesis 1 was supported.

**TABLE 5:** Comparison of outcome variables between the two groups

Variables	Groups	Pre‐test	Post‐test	Difference	*t*/*F*	*p*
		Mean ± SD	Mean ± SD	Mean ± SD		
Cognition of safe handling of antineoplastic drugs						
Total	Exp	102.77 ± 17.23	132.63 ± 9.91	29.86 ± 16.72	6.84	<0.001
	Cont	110.55 ± 16.68	115.60 ± 20.44	5.05 ± 8.93		
Preparing antineoplastic drugs	Exp	46.50 ± 9.09	61.17 ± 4.86	14.67 ± 9.67	5.64	<0.001
	Cont	50.72 ± 9.10	52.70 ± 11.76	1.98 ± 7.59		
Administering antineoplastic drugs	Exp	42.60 ± 7.56	52.67 ± 4.81	10.07 ± 6.80	3.87	<0.001
	Cont	44.10 ± 7.26	46.97 ± 8.50	2.87 ± 7.59		
Disposing of antineoplastic drugs	Exp	13.67 ± 3.64	18.80 ± 1.71	5.13 ± 3.74	31.69^a^	<0.001
	Cont	15.47 ± 3.17	15.90 ± 3.10	0.43 ± 3.75		
Compliance of safe handling of antineoplastic drugs						
Total	Exp	86.75 ± 9.50	110.85 ± 15.88	24.10 ± 15.42	5.86	<0.001
	Cont	81.93 ± 9.47	82.00 ± 8.74	0.07 ± 4.13		
Preparing antineoplastic drugs	Exp	37.16 ± 4.91	48.72 ± 8.05	11.56 ± 6.59	9.42	<0.001
	Cont	34.80 ± 7.00	33.20 ± 5.76	−1.60 ± 4.63		
Administering antineoplastic drugs	Exp	38.62 ± 5.20	46.13 ± 6.12	7.51 ± 7.14	3.74	<0.001
	Cont	37.33 ± 3.68	36.73 ± 3.01	−0.60 ± 3.22		
Disposing of antineoplastic drugs	Exp	12.00 ± 1.92	15.96 ± 2.77	3.96 ± 3.21	4.43	<0.001
	Cont	11.17 ± 1.76	13.95 ± 2.15	2.48 ± 1.40		
The stress handling of antineoplastic drugs						
Total	Exp	46.57 ± 8.65	42.87 ± 11.39	3.70 ± 13.60	5.15	<0.001
	Cont	47.47 ± 11.97	47.49 ± 12.26	0.02 ± 5.50		

^a^ *F*‐test, ANCOVA.

In particular, there were statistically significant results about the cognition of antineoplastic drug disposal. This was analysed using ANCOVA, which showed statistically significant differences in the homogeneity tests, with *F* = 31.69 and *p* < 0.001, indicating the significance.

#### 4.3.2 Hypothesis 2

Hypothesis 2 states that “the experimental group with safe handling education of antineoplastic drugs will have a higher score of practice than the control group”. The findings showed statistically significant results (*t* = 5.86, *p* < 0.001) (Table 5), supporting Hypothesis 2.

#### 4.3.3 Hypothesis 3

Hypothesis 3 states that “the experimental group with the safe handling education of antineoplastic drugs will have a lower score on the stress handling of antineoplastic drugs than the control group”. The findings were statistically significant, and Hypothesis 3 was supported (*t* = 5.15, *p* < 0.001).

## 5 DISCUSSION

The need for increased education has emerged about safe management of anticancer drugs to reduce the risk of exposure to harmful substances by nurses and doctors handling anticancer drugs. This study attempted to understand the effectiveness of education based on the safety principles of anticancer drugs.

### 5.1 Effect on cognition of safe handling education of antineoplastic drugs

In this study, the experimental group's post‐education cognitive scores significantly increased compared to their initial scores. Additionally, there was a significant difference between the experimental and control groups. This is consistent with the results of previous studies (Choi et al., 2004; Keat et al., 2013; Mohsen & Fareed, 2013), verifying the effectiveness of education related to anticancer drugs. The educational program increased awareness about ways to safely handle antineoplastic drugs during their preparation, administration and disposal. There have been instances of caregivers or nurses wearing equipment without utilizing the protective functions or employing incorrect knowledge of safety management principles documented in the past. Therefore, safety management education emphasizes safety management principles and delivers verified knowledge. Nursing practices and the act of wearing of personal protective equipment are important to safely administer anticancer drugs. However, prior to this study they have not been adequately observed. For the safe management of anticancer drugs, it is important for nurses to familiarize themselves with the guidelines for safe handling and the risks associated with mishandling the medication.

### 5.2 Effect on compliance of safe handling education of antineoplastic drugs

In this study, the compliance score for the safe handling of antineoplastic drugs significantly increased in the experimental group post‐education. Additionally, there was a statistically significant difference between the experimental and control groups. This is consistent with the results of previous studies (Chang et al., 2016; Keat et al., 2013; Kiran et al., 2017; Mohsen & Fareed, 2013), verifying the effectiveness of anticancer drug‐related education. In this study, the application of educational programs increased the degree of compliance during preparation, administration and disposal of the medication. Thus, this study concludes that education on the safety management of anticancer drugs is an effective intervention strategy which increases everyday practice. The results imply that education influences behavioural changes regarding safety and effectively warn nurses about the risks associated with exposure to anticancer drugs.

As a consequence of the study conducted by Yun and Park (2016), in 2004, only 58.4% of nurses in the anticancer ward wore personal protective equipment; however, since 2010, this figure increased to more than 90% owing to the evaluation of Korean medical institutions. Safety rules have strengthened, and educational opportunities have increased due to recommendations about hazardous‐substance management and the certification of medical institutions. Therefore, practical implementation of safety management guidelines is essential to minimize exposure to anticancer drugs.

However, according to a review by Simon et al. (2019), the practical implementation of safety management guidelines was not effective in reducing antineoplastic drug contamination. This occurred despite training and proper performance of anticancer drug safety based on safety management guidelines. In other words, current cleaning practices cannot remove residual, trace amounts of drugs from surfaces. These results indicate that the guidelines applied in healthcare settings need to be regularly revised.

Thus, many institutions have revised their previous guidelines in recent years about the safe handling of antineoplastic agents. For example, the Occupational Safety and Health Administration (OSHA) (United States Department of Labor, 2012; Yodaiken & Bennett, 1986) published their initial guidelines on 1986, and updated them in 2012; likewise, the National Institute for Occupational Safety and Health (NIOSH) (2004) published protocols in 2004, and the International Society of Oncology Pharmacy Practitioners (ISOPP) (2007) developed in 2007. Recently, the American Society of Health‐System Pharmacists (ASHSSP) (2018) updated their standards in 2018. To safely handle anticancer drugs and reduce risk of exposure to harmful substances, relevant departments and institutions requires continuous consideration and improvement.

### 5.3 Stress handling of antineoplastic drugs

The post‐education stress score of the experimental group was significantly reduced in this study. This can be understood as a reduction in stress due to a reduced exposure to anticancer drugs prompted by increased cognition of and compliance with anticancer drug safety management principles. Nurses working in environments with many opportunities to be exposed to anticancer drugs may experience psychological stress about the toxic effects of handling anticancer drugs.

Therefore, more robust personal implementation efforts and hospital‐level support in the form of providing appropriate supplies and equipment to encourage of safe practices are required. Similarly, the placement of appropriate nursing personnel for compliance with safety guidelines should be considered (Friese et al., 2019; Polovich, 2016; Walton et al., 2017).

### 5.4 The safe handling education of antineoplastic drugs program

While previous anticancer drug safety management education was conducted as lecture‐centred group education, this study employed both the demonstration and practice of safety principles. Thus, this study contends that 12 h of training over two weeks is more effective than one‐off training in solidifying the cognition of and compliance with anticancer safety management. The effectiveness of anticancer drug safety management education sufficiently tested and demonstrated the preventive effects of individual nurses' cognition and compliance. It is necessary to implement systematic education programs in hospitals and to protect nurses from risks that anticancer drugs pose by preparing systems and policies to comply with anticancer drug safety management rules.

### 5.5 Limitations

This study has several limitations. First, it was carried out with nurses working in local hospitals, and the number of participants was small. Therefore, the results cannot be generalized. It is necessary to verify these effects in larger scale studies, and to repeat these types of studies in different environments in the future. Furthermore, the effectiveness of education programs should be verified at various levels; this study examined general hospitals and future research should examine tertiary hospitals.

Additionally, the educational intervention used in this study was conducted with an emphasis on common anticancer safety management, without accounting for differences between internal medicine and surgical wards. Future research should subdivide and organize the educational content according to the roles and tasks of nurses.

## 6 CONCLUSION

The study results confirmed that education about the safe handling of antineoplastic drugs improved nurses' cognition, compliance and stress handling of antineoplastic drugs. In clinical nursing practice, proper education about the safe management of antineoplastic drugs is expected to contribute to promoting safety management practices and minimizing exposure to antineoplastic drugs.

## AUTHOR CONTRIBUTIONS

Drs. EM Jun and SW Kang were responsible for study design and drafting of the manuscript, and performed data collection and intervention. Dr. SW Kang critically revised and edited the manuscript. There are no conflicts of interest to report.

## CONFLICT OF INTEREST

The authors declare no conflict of interest.

## FUNDING INFORMATION

This work was supported by a research grant from Pai Chai University from 2022.

## ETHICAL APPROVAL

The study was conducted according to the guidelines of the 2022 IRB Dongseo University.

## Data Availability

Data sharing is not applicable to this article as no new data were created or analyzed in this study.
